# Postoperative Gait Characteristics After Arthroscopic Ankle Arthrodesis Using a Deep Learning-Based Markerless Motion Capture System: A Pilot Observational Study

**DOI:** 10.3390/jcm15156034

**Published:** 2026-08-03

**Authors:** Yoon-Chung Sophie Kim, Cesar de Cesar Netto, Youn-Ho Choi, Jae Hoon Ahn

**Affiliations:** 1 Department of Orthopaedic Surgery, St. Vincent’s Hospital, College of Medicine, The Catholic University of Korea, Seoul 06591, Republic of Korea; cruzinbooo@catholic.ac.kr; 2 Department of Orthopaedic Surgery, Duke University, Durham, NC 27710, USA; cesar.netto@duke.edu; 3 Department of Orthopaedic Surgery, Seoul St. Mary’s Hospital, College of Medicine, The Catholic University of Korea, Seoul 06591, Republic of Korea; jahn@catholic.ac.kr

**Keywords:** gait analysis, arthroscopic ankle arthrodesis, deep learning, markerless motion capture, advanced ankle arthritis

## Abstract

**Background/Objectives:** Ankle arthrodesis is an effective treatment for advanced ankle arthritis. Although postoperative gait following open ankle arthrodesis has been investigated, gait characteristics after arthroscopic ankle arthrodesis remain less well characterized. Therefore, we aimed to investigate postoperative gait characteristics after arthroscopic ankle arthrodesis using a deep learning-based markerless motion capture system in this pilot observational study. **Methods:** Seven patients with degenerative ankle arthritis underwent arthroscopic ankle arthrodesis. Real-time gait analysis using a deep learning-based markerless motion capture system assessed gait speed, cadence, step length, stance and swing phases, and ankle range of motion preoperatively and at 1 year postoperatively. Postoperative findings were also compared with those of a minimally symptomatic comparison group. Clinical outcomes were evaluated with the Visual Analog Scale, Foot and Ankle Ability Measure (FAAM), Lower Extremity Functional Scale, American Orthopaedic Foot and Ankle Society Ankle-Hindfoot Scale, and the Karlsson Scale. **Results:** At the 1-year postoperative follow-up, no significant differences in any of the spatiotemporal gait parameters were found between patients and the comparison group. Preoperative ankle range of motion during the gait cycle showed significant deviations from that of the comparison group, which were largely attenuated at the 1-year follow-up, except for persistent plantarflexion restriction during terminal stance. Numerical improvements were observed in most clinical scores, whereas the FAAM-Sports subscale remained essentially unchanged. **Conclusions:** At 1 year postoperatively, spatiotemporal gait parameters did not differ significantly from those of the comparison group, although terminal stance plantarflexion restriction persisted. Markerless motion capture provided objective gait data in a routine clinical setting, but its accuracy requires validation against standard marker-based systems.

## 1. Introduction

Ankle arthritis is a degenerative joint disease characterized by cartilage loss, subchondral bone wear, and osseous remodeling, resulting in chronic pain and functional limitations, including impaired gait [1,2]. Despite the growing role of arthroplasty, ankle arthrodesis (AA) remains the standard treatment for advanced ankle arthritis due to its consistent pain relief and low revision rates [3,4,5]. Traditionally performed via an open approach, arthroscopic AA—introduced in 1983—has demonstrated comparable union and reoperation rates, with fewer complications and shorter hospital stays [6,7,8]. Minimally invasive ankle arthrodesis techniques have also been developed to reduce surgical morbidity while maintaining favorable union and functional outcomes [9].

Given that walking capacity is essential for daily function, gait recovery represents a key therapeutic target following definitive treatment. Prior studies have investigated gait patterns after AA, particularly after open procedures, and have reported gait alterations and compensatory movement at adjacent joints [10]. However, because previous gait studies have largely involved open procedures [11], objective longitudinal gait characterization after arthroscopic AA remains comparatively limited, despite the availability of studies reporting conventional clinical and radiographic outcomes [7,8].

To assess gait change after arthroscopic AA, we employed objective gait analysis using a deep learning-based markerless motion capture system. This technology enables real-time analysis from simple video recordings in routine clinical environments, instead of dedicated laboratory settings, and eliminates the need for reflective markers that may interfere with natural walking [12]. This approach allows repeated, noninvasive evaluations and provides both spatiotemporal data and joint kinematics, reflecting more natural gait in real-world settings. Building on this capability, this preliminary study investigated postoperative gait characteristics after arthroscopic AA alongside conventional clinical and radiographic outcomes.

## 2. Materials and Methods

### 2.1. Patient Selection

**Figure 1:**
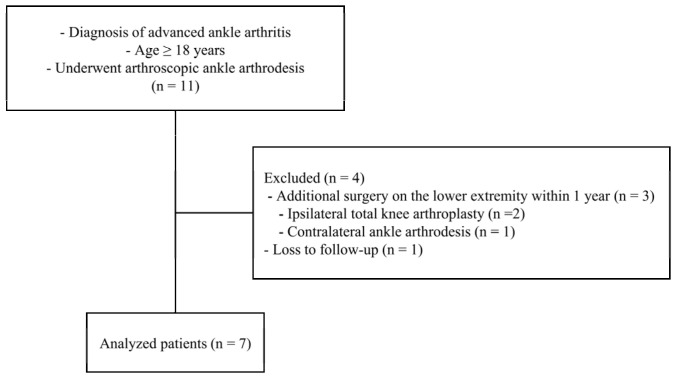
Flowchart showing the selection of patients.

This retrospective observational study included patients who underwent arthroscopic AA at our hospital between December 2022 and October 2023. Arthroscopic AA was indicated for advanced ankle arthritis refractory to nonoperative treatment when adequate joint preparation and deformity correction were considered achievable arthroscopically [1]. The study was approved by our institutional review board and was reported in accordance with the Strengthening the Reporting of Observational Studies in Epidemiology (STROBE) statement. To contextualize the precision achievable in this retrospective pilot study, gait speed was selected as a representative outcome variable because of its established clinical relevance. Previous reports in lower-limb osteoarthritis have shown a standard deviation of approximately 0.20 m/s [13], and a moderate clinically important change in gait speed has been reported as approximately 0.14 m/s [14]. Considering the feasibility and exploratory nature of the study, a precision-based calculation was performed using gait speed as the representative outcome variable [15]. The calculation used the following formula:

(1) $$n={\left(\frac{Z_{1-\alpha /2}\sigma }{d}\right)}^{2}$$

where *Z*_1−α/2_ = 1.96 for a 95% confidence level, σ = 0.20 m/s, and the desired confidence interval half-width *d* = 0.14 m/s. During the study period, seven eligible patients were available, and all were included in this exploratory pilot study. Details of the inclusion and exclusion criteria are summarized in Figure 1. For comparison, a minimally symptomatic comparison group providing reference gait data was established, consisting of twenty outpatients with mild hallux valgus, defined as a hallux valgus angle less than 20 degrees and minimal pain during ambulation, indicated by a Visual Analog Scale (VAS) score of 1 or lower. Previous studies have shown that even symptomatic hallux valgus patients exhibit no substantial differences in spatiotemporal gait parameters compared with asymptomatic controls [16,17], supporting the use of this population as a comparison group in this study.

### 2.2. Outcome Measures

Patients in the operated group underwent gait, clinical, and radiographic assessments, whereas participants in the comparison group underwent gait assessment. Gait analysis was conducted using real-time, deep learning-assisted, markerless motion capture software (REMOBody-S, version 1.8, REMO Inc., Asan-si, Republic of Korea) based on the Human Motion and Shape Prior (HuMoR) architecture. The HuMoR was used to estimate the three-dimensional positions of twenty-two body keypoints from red-green-blue (RGB) images obtained with a single camera [18]. Because markerless motion capture can be influenced by camera viewing angle and distance [19], all participants walked back and forth along a 3-m straight path, with the camera fixed at a 45-degree angle and 5 m from the path to standardize data acquisition. The same acquisition protocol was used at each time point to reduce measurement variability. Gait analysis was performed preoperatively and at 1 year postoperatively to assess changes in nine spatiotemporal gait parameters and ankle range of motion (ROM). Spatiotemporal parameters included gait speed, cadence, step length, stance phase, swing phase, double support phase, opposite heel strike, cycle duration, and step width. Gait data were automatically generated and stored by the REMOBody-S system. One investigator exported the data and matched them to the patient and assessment time point, and a second investigator cross-checked the final dataset against the original output files. For gait parameter analysis, three complementary approaches were used to enhance clinical relevance. First, mean differences in gait parameters were statistically compared between the operated limb and the comparison group at the 1-year follow-up. Second, interlimb differences were descriptively examined rather than formally tested. The 95% limits of agreement (LoA) derived from the comparison group were used as a reference range for determining whether postoperative interlimb asymmetry exceeded the variability observed in the comparison group. The 95% LoA were calculated as the mean interlimb difference ±1.96 standard deviations of the interlimb differences. Values outside these limits were considered to indicate asymmetry greater than the reference range. Third, preoperative and 1-year postoperative gait parameters of the operated limb were directly compared to evaluate within-patient changes. Ankle kinematics were also assessed using statistical parametric mapping (SPM) to compare pre- and postoperative values to those of the comparison group [20]. SPM analyses were performed using Python 3.14.2 with spm1d 0.4.53. Kinematic trajectories were smoothed using a Gaussian kernel with a full width at half maximum of 5 samples. Statistical significance was set at α = 0.05 with family-wise error correction.

Clinical evaluation included the VAS, Foot and Ankle Ability Measure (FAAM), Lower Extremity Functional Scale (LEFS), American Orthopaedic Foot and Ankle Society (AOFAS) Ankle-Hindfoot Scale, and the Karlsson Scale. These clinical scores were obtained one day before surgery and at 1 year postoperatively. Pre- and postoperative scores were compared to evaluate clinical improvement. Clinical scores were extracted from routine medical records by one investigator and independently verified by another investigator.

Radiographic assessment was performed using standing ankle radiographs to evaluate bony union at the fusion site. Standing radiographs were obtained preoperatively and at 3, 6, 9, and 12 months postoperatively to assess the progression of bony union. Bony union was defined as radiographic evidence of osseous bridging by 6 months postoperatively, accompanied by painless ambulation without assistive devices in daily life [21]. Nonunion was defined as the absence of radiographic union at 6 months or persistent pain impairing ambulation. Union status was determined by an independent investigator blinded to surgical procedures. Time to union was recorded for each patient. Other complications were investigated, including wound infections, fixation failure requiring revision surgery, or thromboembolic complications. All clinical scores and radiographic data were obtained from the hospital electronic medical record system and picture archiving and communication system, respectively. All seven patients completed the scheduled assessments, with no missing data.

### 2.3. Operative Technique and Postoperative Care

All procedures were performed by a single fellowship-trained foot and ankle surgeon with prior experience in ankle arthroscopy and arthroscopic arthrodesis. Surgery was performed under general anesthesia with the patient in the lateral decubitus position. Ankle arthroscopy was performed under non-invasive distraction without the use of a tourniquet. The distraction system secured the thigh with the hip and knee flexed to 90 degrees, and the foot with a resterilizable strap [22]. A 2.7- or 2.4-mm 30° arthroscope was introduced via anteromedial, anterolateral, and posterolateral portals; accessory portals were created as necessary. Synovectomy, cartilage debridement, and subchondral preparation were performed throughout the ankle joint, including the medial and lateral gutters, with emphasis on the talofibular articulation.

Under fluoroscopic guidance, the talar dome and distal tibia were aligned to correct talar varus and anterior translation. Previous studies have shown that a fibular onlay strut graft can enhance rotational and lateral stability by providing lateral support to maintain neutral alignment and reduce the risk of valgus deformity [23,24]. Based on this concept, temporary fixation with a Kirschner wire was followed by placement of a 6.5-mm headless compression screw directed through the fibula to secure the talus against it, thereby achieving talofibular compression and providing additional lateral support, contributing to varus correction and lateral stability. Two additional headless compression screws were then inserted from conventional trajectories, resulting in three screws in total. After confirming stable fixation, the wounds were closed and a short-leg splint was applied.

**Figure 2:**
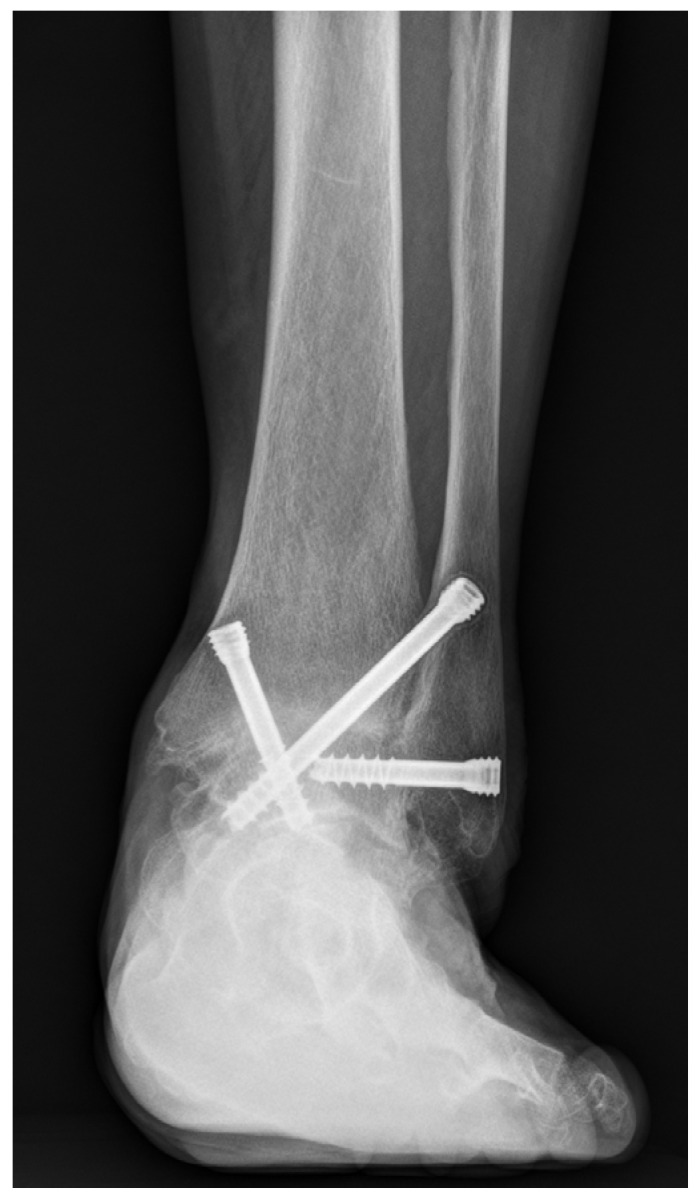
Six-month follow-up plain radiographs demonstrating complete bony union along the arthrodesis site.

The ankle was maintained in a neutral position using a short-leg splint. On postoperative day 7, sutures at the arthroscopic portal and screw sites were removed, and a short-leg cast was applied. Patients were discharged with instructions for non-weight-bearing ambulation using crutches or a wheelchair for 6 weeks. At 8 weeks, the cast was removed and protected weight-bearing was initiated with a controlled ankle motion boot fixed at 90 degrees. Walking aids were gradually discontinued, and by 12 weeks, patients were allowed to wear regular shoes and begin self-ambulation. Sports and heavy loading were restricted until 6 months, following radiographic confirmation of bony union (Figure 2).

### 2.4. Statistical Analysis

For clinical outcomes and other continuous variables, comparisons were performed using Student’s *t*-test or Mann–Whitney U-test, as appropriate, and categorical variables were compared using Fisher’s exact test. All statistical analyses were performed by Y.H.C., one of the study investigators, using R software (ver. 4.2.1; R Foundation for Statistical Computing, Vienna, Austria). A *p*-value < 0.05 was considered statistically significant. Preoperative and postoperative gait parameters were compared using paired *t*-tests. *p*-values for the nine spatiotemporal gait parameters in each comparison were adjusted separately using the Benjamini–Hochberg false discovery rate method. The six clinical outcome *p*-values were adjusted separately using the same method.

## 3. Results

### 3.1. Gait Parameters and Ankle Joint Kinematics

The baseline demographic and clinical data for all patients are shown in Table 1. At the 1-year follow-up, there were no statistically significant differences in any of the nine spatiotemporal gait parameters between the operated limb of the patient group and the comparison group. Furthermore, no patient had an interlimb difference outside the 95% LoA derived from the comparison group (Table 2). Gait speed and step length increased significantly from preoperatively to 1 year postoperatively. However, neither remained statistically significant after false discovery rate correction (Table 3).

**Figure 3:**
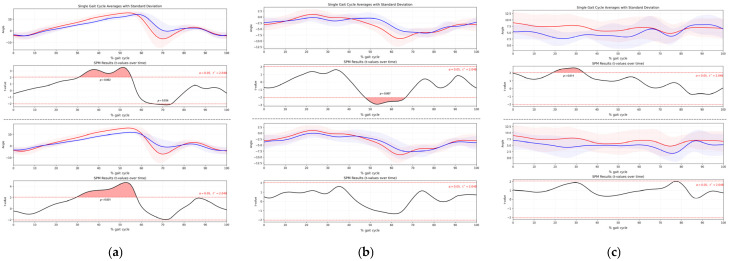
Changes in ankle joint range of motion (ROM) throughout the gait cycle, assessed using statistical parametric mapping (SPM), are shown for the operated limb (blue line) and the comparison group (red line). Each panel includes mean gait waveforms (**top**) and corresponding SPM *t*-value curves (**bottom**), with shaded areas representing standard deviations. The upper two graphs present preoperative data, while the lower two graphs display data at 1 year postoperatively. Red dashed horizontal lines indicate the positive and negative critical t-statistic thresholds (±t*) for SPM inference at α = 0.05, and red-shaded areas indicate intervals in which the SPM t-value curve exceeded the positive threshold or fell below the negative threshold, denoting statistically significant differences between groups. Three axes of ankle motion are illustrated: (**a**) dorsiflexion and plantarflexion; (**b**) eversion and inversion; and (**c**) abduction and adduction.

**Figure 4:**
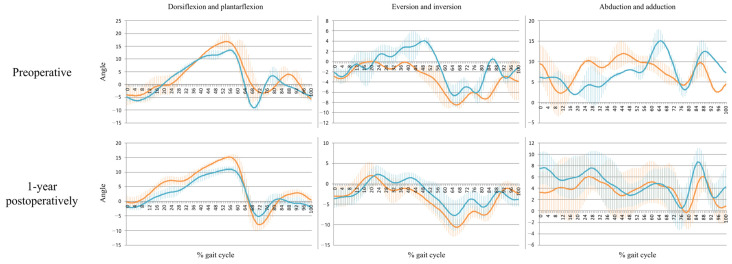
Representative preoperative and 1-year postoperative ankle kinematic curves of an individual patient throughout the gait cycle. The blue and orange lines represent the operated and contralateral limbs, respectively.

Preoperative ankle ROM during the gait cycle showed statistically significant deviations from those of the comparison group across all three axes (sagittal, coronal, and axial). Postoperatively, the differences in coronal and axial kinematics were no longer significant. However, due to the inherent nature of AA, plantarflexion restriction persisted during terminal stance (Figure 3). Figure 4 presents the preoperative and 1-year postoperative kinematic curves of a representative patient from the study cohort. At the individual level, the operated and contralateral limbs showed greater convergence in coronal and axial kinematic patterns postoperatively than preoperatively, while reduced plantarflexion during terminal stance persisted in the sagittal plane, consistent with the group-level findings. In this patient, gait speed increased from 0.68 to 0.73 m/s, cadence increased from 108.09 to 113.85 steps/min, and step length increased from 37.56 to 38.64 cm. Step width decreased from 23.65 to 17.62 cm, while the proportions of the stance and swing phases remained essentially unchanged. Clinical outcomes also improved, with the VAS score decreasing from 7 to 3. The FAAM activities of daily living (ADL) and sports subscale scores increased from 50.2 to 65.3 and from 35.7 to 41.4, respectively. The LEFS, AOFAS Ankle-Hindfoot Scale, and Karlsson Scale scores increased from 27 to 45, 28 to 74, and 20 to 32, respectively.

### 3.2. Clinical and Radiographic Outcomes

At 1 year postoperatively, VAS and AOFAS Ankle-Hindfoot Scale scores improved significantly before correction. However, neither remained statistically significant after false discovery rate correction (Table 4). All other clinical scores, with the exception of the FAAM-Sports subscale, also improved, though without reaching statistical significance. All patients achieved bony union within 6 months based on the predefined criteria, and no cases of delayed union were observed. The mean time to union was 5.6 ± 1.1 months (range, 3–6 months). No postoperative complications, including nonunion, wound infection, fixation failure, or thromboembolic events, were observed.

## 4. Discussion

This pilot observational study characterized gait patterns 1 year after arthroscopic AA and found no statistically significant differences in spatiotemporal parameters between the operated and comparison groups, while terminal stance plantarflexion restriction persisted, as expected after fusion. This finding is relevant because postoperative gait after arthroscopic AA remains less well characterized than gait after AA in general, and most available studies have focused on open techniques [10,11]. Given this gap in the literature, we sought to characterize postoperative gait patterns in greater detail. To provide clinically interpretable results, gait parameters were analyzed using three complementary approaches. First, we compared the operated limbs at 1 year postoperatively with those of the comparison group and found no statistically significant differences. Second, we descriptively compared postoperative interlimb differences with the 95% LoA derived from the comparison group to determine whether asymmetry exceeded the interlimb variability observed in the comparison group. The absence of interlimb differences outside this reference range is consistent with previous reports demonstrating preserved gait symmetry following tibiotalar arthrodesis, despite complete fusion of the ankle joint. Prior in vivo imaging studies have suggested that compensatory motion at the subtalar joint may contribute to maintaining gait symmetry after AA [25]. Third, direct comparison of the operated limb between the preoperative and 1-year postoperative assessments showed significant increases in gait speed and step length before adjustment. However, neither remained statistically significant after false discovery rate correction.

In our cohort, although sagittal plane ankle ROM remained reduced, postoperative differences in coronal and axial kinematics relative to the comparison group were no longer statistically significant. This finding should not be interpreted as restoration of motion at the fused tibiotalar joint. Rather, it may suggest favorable changes in overall ankle kinematics, particularly in the coronal and axial planes, despite persistent sagittal plane restriction. One possible explanation is that pain relief and improved functional stability following arthroscopic AA may have allowed more physiologic motion at adjacent joints, including the subtalar joint, which is known to contribute primarily to inversion-eversion and internal-external rotation during gait [26,27]. This may have reduced preoperative abnormal or protective movement patterns and contributed to the postoperative coronal and axial kinematic patterns observed. However, because the foot was modeled as a single rigid segment, the measured kinematics represented overall ankle motion and could not isolate subtalar joint motion. Therefore, any interpretation of subtalar compensation remains speculative, and future studies using multisegment foot models or dual fluoroscopy imaging are warranted. Although no statistically significant postoperative differences were observed in coronal and axial kinematics between patients and the comparison group, it is possible that these findings reflect limitations in measurement precision or repeatability in these planes. Notably, the relatively wide standard deviations in axial parameters (Figure 3c), consistent with known limitations of traditional marker-based three-dimensional motion analysis in non-sagittal planes [28,29], may have obscured subtle group-level differences. In addition, the small sample size limited the statistical power to detect postoperative differences. The lack of concurrent validation against marker-based motion capture further limits the reliability and precision of these findings.

A key methodological feature of this study was the incorporation of markerless gait analysis into routine outpatient assessment. Unlike conventional marker-based systems, which require specialized equipment, personnel, and dedicated laboratory space [30,31], the single-camera HuMoR-based system enabled practical acquisition of spatiotemporal and kinematic data in a clinical setting [12,18]. Its ease of setup and portability facilitated objective assessment of dynamic gait characteristics that cannot be captured by patient-reported outcomes or static imaging alone [32,33]. Given the clinical importance of gait recovery after foot and ankle surgery [34], this setup may facilitate objective assessment during routine postoperative follow-up, although further validation against standard marker-based systems is required.

In this cohort, VAS and AOFAS Ankle-Hindfoot Scale scores improved before adjustment for multiple comparisons, although neither remained statistically significant after false discovery rate correction. The other clinical outcomes did not show statistically significant changes, and the FAAM-Sports subscale remained essentially unchanged, suggesting that limitations in higher-demand activities may persist despite numerical improvements in pain and basic function. All patients achieved bony union, and no postoperative complications were observed. These findings are encouraging and are consistent with previous reports of favorable outcomes after arthroscopic AA [7,8].

In our technique, the concept of a fibular onlay graft was applied to provide additional lateral support and assist alignment during fixation, based on previous reports describing its biomechanical contributions in AA [35]. The cancellous surface of the fibula, when adequately prepared, may also offer a biologically favorable environment for bone healing [24,36], which may have contributed to the timely union observed in all patients. Previous minimally invasive ankle arthrodesis studies have also reported favorable union, complication, and functional outcomes [9], although direct comparison with our findings is limited by differences in surgical technique and study design. Overall, arthroscopic AA with this adjunct appeared to provide a stable fusion environment in this cohort. However, the observed 100% union rate and absence of complications should be interpreted cautiously because the small sample size results in considerable uncertainty around these estimates and precludes firm conclusions regarding the specific contribution of the adjunct or procedural safety.

This study has several limitations. First, the sample size was relatively small, reflecting the preliminary nature of this pilot observational study. The limited enrollment reflects the relatively low number of arthroscopic AA procedures performed during the study period and the fact that markerless motion capture is still infrequently used in clinical research, where studies with similarly small cohorts have nevertheless provided meaningful insights [37,38]. Nevertheless, the small sample size may have limited statistical power and increased the risk of type II error. Therefore, the findings should be interpreted cautiously and should not be generalized to broader patient populations. Second, gait was assessed only preoperatively and at 1 year postoperatively, limiting our ability to evaluate the temporal course of postoperative gait recovery. In addition, the follow-up duration was limited to one year. Future studies with serial assessments at multiple postoperative time points and longer-term follow-up are needed to characterize recovery trajectories and evaluate the durability of surgical outcomes and postoperative gait patterns. Third, the comparison group consisted of patients with mild hallux valgus rather than matched healthy volunteers, and differences in demographic characteristics were observed between the groups. Therefore, comparisons between the groups should be interpreted with caution. Fourth, the repeatability and validity of the markerless motion capture system were not evaluated in patients undergoing ankle arthrodesis, and the system was not concurrently compared with a marker-based three-dimensional motion capture system. Therefore, measurement error may have influenced the observed lack of differences, and the findings should be interpreted as preliminary. Fifth, because this was a retrospective observational study, causal relationships between arthroscopic AA and postoperative gait changes could not be established. Sixth, the retrospective selection of patients with complete 1-year gait data may have introduced selection bias, and potential confounders such as rehabilitation adherence, physical activity level, analgesic use, and baseline disease severity were not controlled. Given the small sample size, meaningful multivariable analyses to adjust for these potential confounders could not be performed.

## 5. Conclusions

In this pilot observational study, no statistically significant differences in postoperative spatiotemporal gait parameters were observed between patients undergoing arthroscopic AA and the comparison group, although terminal stance plantarflexion restriction persisted. Under its known limitations, the deep learning-based markerless motion capture system provided objective gait data in routine clinical settings and demonstrated preliminary clinical feasibility. However, its accuracy requires further validation against standard marker-based three-dimensional motion capture systems. Larger prospective studies with longer follow-up and standardized control populations are needed to confirm these findings.

## Figures and Tables

**Table 1:** Characteristics of the patients included in this study.

Variables	Operated Group	Comparison Group	*p*-Value
Demographic data			
Patients	7	20	
Age (years)	63.0 ± 10.3	58.0 ± 11.5	0.306
Female	2 (28.6%)	18 (90.0%)	0.005
Weight (kg)	64.9 ± 10.4	58.4 ± 12.6	0.202
BMI (kg/m^2^)	27.0 ± 3.24	25.8 ± 3.01	0.411
Underlying disease			
Diabetes	2 (28.6%)	3 (15.0%)	0.580
Coronary artery disease	1 (14.3%)	1 (5.0%)	0.459
Cerebrovascular accident	0 (0%)	0 (0%)	NA *
Kidney disease	1 (14.3%)	1 (5.0%)	0.459

BMI, Body mass index; NA, Not applicable. * Statistical comparison was not applicable because no events occurred in either group.

**Table 2:** Comparison of gait parameters between the patients and the comparison group at the 1-year follow-up.

Parameters	Operated Limb	Comparison Group	*p*-Value (Adjusted *p*-Value *)	Difference Between Operated and Non-Operated Limb			Interlimb Difference (95% LoA, Comparison Group)
				Mean	Min	Max	
Gait speed (m/s)	0.78 ± 0.13	0.85 ± 0.14	0.122 (0.516)	0.03 ± 0.05	−0.04	0.08	−0.13 to 0.17
Cadence (steps/min)	105.2 ± 6.00	102.9 ± 8.84	0.609 (0.685)	2.20 ± 2.81	−2.21	5.43	−13.3 to 10.8
Step length (cm)	44.6 ± 6.81	44.7 ± 6.16	0.750 (0.750)	1.09 ± 3.64	−4.96	5.39	−7.88 to 7.03
Stance phase (%)	69.1 ± 3.45	67.4 ± 5.02	0.249 (0.516)	−1.09 ± 0.85	−2.4	−0.14	−4.75 to 4.31
Swing phase (%)	30.9 ± 3.45	32.6 ± 5.02	0.249 (0.516)	1.09 ± 0.85	0.14	2.4	−4.31 to 4.75
Double support phase (%)	19.4 ± 1.86	17.1 ± 4.81	0.401 (0.516)	1.46 ± 2.40	−1.55	3.41	−6.17 to 4.13
Opposite heel strike (%)	49.1 ± 2.02	49.6 ± 1.85	0.211 (0.516)	0.86 ± 1.81	−1.56	3.15	−5.15 to 3.25
Cycle duration (s)	1.14 ± 0.11	1.17 ± 0.10	0.397 (0.516)	0.00 ± 0.03	−0.04	0.06	−0.13 to 0.12
Step width (cm)	19.6 ± 2.12	18.4 ± 3.26	0.395 (0.516)	0.43 ± 4.57	−6.47	3.92	−7.40 to 6.42

LoA, limits of agreement. * Values in parentheses are *p*-values adjusted using the Benjamini–Hochberg false discovery rate method.

**Table 3:** Comparison of preoperative and 1-year postoperative spatiotemporal gait parameters.

Parameters	Preoperative	1 Year After Surgery	Mean Change (95% CI)	*p*-Value * (Adjusted *p*-Value ^†^)
Gait speed (m/s)	0.73 ± 0.14	0.78 ± 0.13	+0.05 (0.001 to 0.097)	0.034 (0.153)
Cadence (steps/min)	107.7 ± 4.27	105.2 ± 6.00	−2.5 (−7.25 to 2.21)	0.229 (0.468)
Step length (cm)	41.7 ± 7.30	44.6 ± 6.81	+2.9 (0.818 to 4.83)	0.015 (0.135)
Stance phase (%)	69.7 ± 1.65	69.1 ± 3.45	−0.6 (−3.73 to 2.58)	0.658 (0.658)
Swing phase (%)	30.3 ± 1.65	30.9 ± 3.45	+0.6 (−2.58 to 3.73)	0.658 (0.658)
Double support phase (%)	19.7 ± 1.31	19.4 ± 1.86	−0.3 (−2.18 to 1.50)	0.651 (0.658)
Opposite heel strike (%)	48.2 ± 1.96	49.1 ± 2.02	+0.9 (−0.961 to 2.83)	0.260 (0.468)
Cycle duration (s)	1.11 ± 0.04	1.14 ± 0.11	+0.03 (−0.049 to 0.099)	0.422 (0.633)
Step width (cm)	18.0 ± 1.79	19.6 ± 2.12	+1.6 (−1.23 to 4.39)	0.209 (0.468)

CI, Confidence interval. * *p*-values were calculated using paired *t*-tests. ^†^ Values in parentheses are *p*-values adjusted using the Benjamini–Hochberg false discovery rate method.

**Table 4:** Changes in clinical scores from baseline to the 1-year follow-up.

Variables	Preoperative	1 Year After Surgery	*p*-Value (Adjusted *p*-Value *)
VAS	6.00 ± 2.16	3.00 ± 2.00	0.022 (0.066)
FAAM-ADL subscale	63.8 ± 15.2	67.0 ± 7.37	0.680 (0.816)
FAAM-Sports subscale	34.6 ± 14.7	33.8 ± 17.9	0.910 (0.910)
LEFS	41.1 ± 11.1	48.0 ± 10.2	0.211 (0.317)
AOFAS Ankle-Hindfoot Scale	43.7 ± 16.9	70.7 ± 14.3	0.011 (0.066)
Karlsson Scale	33.1 ± 19.5	49.4 ± 17.7	0.118 (0.236)

VAS, Visual Analog Scale; FAAM, Foot and Ankle Ability Measure; ADL, Activities of Daily Living; AOFAS, American Orthopaedic Foot and Ankle Society; LEFS, Lower Extremity Functional Scale. * Values in parentheses are *p*-values adjusted using the Benjamini–Hochberg false discovery rate method.

## Data Availability

The data presented in this study are available on request from the corresponding author due to privacy and ethical restrictions.

## Abbreviations

The following abbreviations are used in this manuscript:

AA	Ankle arthrodesis
VAS	Visual Analog Scale
HuMoR	Human Motion and Shape Prior
RGB	Red-green-blue
ROM	Range of motion
LoA	Limits of agreement
FAAM	Foot and Ankle Ability Measure
LEFS	Lower Extremity Functional Scale
AOFAS	American Orthopaedic Foot and Ankle Society
BMI	Body mass index
SPM	Statistical parametric mapping
